# Gender differences in the prevalence of anxiety and post-traumatic stress disorder and associated factors among adults in sub-Saharan Africa: a systematic review and meta-analysis

**DOI:** 10.3389/fpsyt.2026.1817602

**Published:** 2026-07-23

**Authors:** Basil M. Masinza, Amina Abubakar, Joy S. Mukubuyi, Patrick N. Mwangala

**Affiliations:** 1 Institute for Human Development, Aga Khan University, Nairobi, Kenya; 2 Centre for Geographic Medicine Research Coast, Kenya Medical Research Institute (KEMRI), Kilifi, Kenya; 3 Department of Public Health, Pwani University, Kilifi, Kenya

**Keywords:** gender differences, anxiety, post traumatic, stress disorder, adults, sub-Saharan Africa

## Abstract

**Background:**

Sex and gender equity in research has a growing focus, but there are gaps in fully integrating it in mental health research, limiting adoption of sex and gender-sensitive interventions. This review adopted a gendered perspective to synthesize prevalence and associated factors contributing to differences in anxiety and PTSD in SSA.

**Methodology:**

Electronic search was conducted in PubMed, PsycINFO, CINAHL, Scopus, Web of Science databases. Empirical studies conducted in SSA, targeting adults >18 years and having gender-disaggregated anxiety and PTSD prevalence and factors were included. Meta-analysis of proportions stratified by sex was done, and narrative synthesis of the significant factors in the multivariable analysis adopted.

**Results:**

From 175 studies included, 75 reported anxiety, 86 reported PTSD, while 14 reported both anxiety and PTSD outcomes. Eastern Africa had 61% representation, 19% in West Africa, 18% in Southern Africa and 2% in Central Africa. High anxiety (36%) and PTSD (34%) prevalence was noted regardless of gender. Women had 39% pooled anxiety prevalence, compared to 30% in men. PTSD prevalence was also higher in women (39%) than men (26%). Women had significantly higher odds of anxiety (Female: Male OR-1.52) and PTSD (Female: Male OR-1.59) in SSA and in the regional analysis. Majority of the factors noted were focused on women. Demographic factors and traumatic experiences were majorly noted in men, with women having a range of factors- demographic, interpersonal, traumatic experiences, and obstetric factors that contributed to the burden.

**Conclusion:**

Anxiety and PTSD are highly prevalent in SSA, women more affected. Although gender differences in the prevalence was well documented across different studies, there is still a paucity of research looking into the factors contributing to the disparities. It is crucial to look further into the modifiable factors that drive the disparities in order to guide targeted, gender-sensitive interventions and mental health programs.

**Systematic Review Registration:**

https://osf.io/dashboard, identifier https://doi.org/10.17605/OSF.IO/NX4JA.

## 1 Introduction

Sex and gender equity in research has had a growing focus, with global institutions like World Health Organization (WHO) (1), Canadian Institutes of Health Research (CIHR) (2), United States National Institute of Health, European Commission Directorate-General for Research and Innovation (3), and Intergovernmental Authority on Development (IGAD) (4) pushing for guidelines informing its inclusion in research, and emphasizing the use of sex and gender-sensitive data and indicators. However, there is still laxity in integrating sex and gender in mental health research globally (5). This oversight leads to continued gender bias, limits presentation of health concerns, and adoption of sex- and gender-sensitive interventions. Failure to clearly note the impact of sex and gender undermines equitable research (5). With the rise of equity-related discussions in health policy, there is a global call to include gender in mental health research impact assessment by funders, researchers, and institutions.

Sex and gender interact in complex ways that affect health. The interaction of biological and social vulnerability contributes to the differences in prevalence, expression, and management of mental health conditions in males and females. Sex differences in the prevalence of these disorders vary with age and the interplay with different environmental exposures (6). Generally, women tend to have more internalizing disorders like anxiety and depression, while men have more externalizing disorders, like substance abuse and antisocial personality (7). In childhood and adolescence, boys have a higher prevalence of conduct disorders, compared to disorders related to body image and self-esteem, like depression and eating disorders in girls (8). Globally, depression and anxiety have been identified to have a higher prevalence in women (9). These mental health patterns result from socially constructed gender roles, power imbalance, and biological differences, all influencing the nature of disorders and health-seeking behaviors (9). For example, different roles and responsibilities place women in positions with little say over certain choices, creating a sense of lack of autonomy, with men having issues relating to socialization and expressing their concerns and emotions (9). All these shape differences in resilience (10), reporting, and identifying the true burden of CMDs globally. This calls for a comprehensive view and understanding of the differences in mental health disorders from a multifaceted approach (11).

Common mental disorders (CMDs) are highly prevalent globally, anxiety and depression being the most common. Anxiety disorders cause a significant burden in different regions, with some conflicting sex differences noted in studies conducted in various settings and populations. Over the past few decades, there has been a growing research focus on common mental disorders. Empirical studies conducted in different sub-Saharan African regions, and investigating diverse populations, have documented the prevalence and burden of these disorders. The reviews (12, 13) and studies have noted different findings with the experience and prevalence of anxiety and PTSD in men and women. A review conducted among Internally Displaced People (IDPs) in SSA reported female gender as a significant determinant contributing to PTSD prevalence (12). During the COVID-19 pandemic, a review in Africa noted higher anxiety prevalence among women (14). However, not all studies reported consistent sex differences. For instance, among adolescents in Nigeria, one study noted higher prevalence of anxiety in boys (32.1%) compared to girls (21.9%) (15). Similarly, a study among South African university students, using a psychometric measurement approach to determine whether sex contributes to the differences in measurement of anxiety reported no significant difference in prevalence and symptom presentation (16).

Differences in the stressors may contribute to the numbers seen. A study investigating PTSD among displaced people in Zimbabwe reported no significant difference in pre and post-migration post-traumatic stress symptoms in men and women, though women had significantly higher effect sizes in the stressors (17). Different forms of stressors were noted in the study, women reporting more of physical harassment, and men noting life threats and lack of shelter to stay as major stressors. A recent study reported that women have an increased likelihood of experiencing sexual assault and abuse (18), and of developing acute psychological disorders characterized by intrusive memories after trauma (19). Furthermore, women’s’ inherent low tolerance for stress might make them vulnerable to this condition, in addition to hormonal differences that significantly impact PTSD (18). A review on social and cultural diversities in stress-related issues noted that women are more subject to sexual and interpersonal trauma, while men more often experience physical, accidental, and assaultive experiences. These differences, largely, are a product of societal and cultural norms that dictate distinct roles (20).

A multi-country study conducted in Uganda, Syria, Rwanda, and Sri Lanka reported high prevalence of PTSD in some contexts, and found no significant increased PTSD prevalence in women when accounting for trauma load, especially in war-affected populations where all genders experience similar stressors (21). These varying results highlight conflicting evidence with gender differences in anxiety and PTSD prevalence. Some studies report higher prevalence in women, others report no significant differences, or higher prevalence in men in different contexts.

Of concern in SSA is the limited adoption of a gender approach in mental health research, and specifically in anxiety and PTSD. Most studies generalize findings (22, 23), or focus on specific populations and countries (24, 25). Reviews done between 2014 and 2024 have addressed specific countries or global trends, and often lack gender-stratified analysis (12, 18, 26–31). A 2020 review presented gender-stratified PTSD data (32) covering 10 countries, and noted a limitation in regional coverage. The general lack of synthesis of gender-focused data limits the provision of gender-specific actions. This review adopts a gendered perspective to document the prevalence and associated factors of anxiety and PTSD among adults in sub-Saharan Africa, noting the geographical patterns, thus providing researchers, practitioners, and policymakers with gender-specific data to inform focused research and initiatives. The specific objectives of this review were:

1. To document the gender-stratified prevalence of Generalized Anxiety Disorder (GAD) and PTSD among adults in Sub-Saharan Africa.
2. To identify the gender-stratified correlates of GAD and PTSD among adults in Sub-Saharan Africa.

## 2 Methodology

### 2.1 Review protocol and registration

This systematic review was guided by a protocol that was developed and registered in the Open Science Framework on 12th May 2025 (https://doi.org/10.17605/OSF.IO/NX4JA). The design and reporting of the systematic review was informed by the Preferred Reporting Items for Systematic Review and Meta-analysis (PRISMA guidelines) (33).

### 2.2 Data sources

The structured electronic searches were conducted in five online databases: PsycINFO, PubMed (MEDLINE), CINAHL, Scopus, and Web of Science. We also searched through the reference lists of included articles (and any relevant systematic review) and the digital archives of Africa Journals Online to access additional articles. The first search was done on 16^th^ February and updated on 16^th^ May 2025. The search was restricted to English and human studies done in sub-Saharan African population. Deduplication and screening was done using Systematic Review Accelerator tool (34). All the retrieved references were exported and managed in EndNote (version X9).

### 2.3 Search strategy

A pre-developed search strategy with the key terms; “anxiety/post-traumatic stress disorder”, “adult”, “sub-Saharan Africa”, combined by the Boolean operator “AND” was used in the electronic search. Synonyms for each key term were combined using the Boolean operator “OR”. Medical Subject Headings (MeSH) terms were applied in PUBMED, PsycINFO and CINAHL database searches. **Table 1** shows the keywords and the synonyms used in the search.

**Table 1:** Search strategy.

Keyword	Synonyms
Anxiety/posttraumatic stress disorder	Anxiety OR Anxiety Disorders OR Anxi* OR Anxiety symptoms OR Symptoms of anxiety OR Generalized anxiety disorder OR GAD OR posttraumatic stress disorder OR PTSD OR Traumatic stress OR Post-traumatic stress OR trauma OR psychological trauma OR Stress disorders
AND	
Adult	Adult* OR old age OR elder*
AND	
sub-Saharan Africa	Africa South of the Sahara OR Sub Saharan Africa OR sub-Saharan Africa OR Sub-Saharan Africa OR Africa OR Angola OR Benin OR Botswana OR Burkina Faso OR Upper Volta OR Burundi OR Cameroon OR Cape Verde OR Central African Republic OR Chad OR Comoros OR Congo OR Cote D’ivoire OR Ivory Coast OR Zaire OR Democratic Republic Of The Congo OR French Somaliland OR Djibouti OR Equatorial Guinea OR Eritrea OR Ethiopia OR Gabonese Republic OR Gabon OR Gambia OR Gold Coast OR Ghana OR Guinea OR Guinea-Bissau OR Kenya OR Basutoland OR Lesotho OR Liberia OR Malagasy Republic OR Madagascar OR Nyasaland OR Malawi OR Mali OR Mauritania OR Mauritius OR Mayotte OR Mozambique OR Namibia OR Niger OR Nigeria OR Reunion OR Rwanda OR Ruanda-Urundi OR Sao Tome & Principe OR Sao Tome OR Senegal OR Seychelles OR Sierra Leone OR Somalia OR South Africa OR South Sudan OR Sudan OR Swaziland OR Eswatini OR Togolese Republic OR Togo OR Uganda OR United Republic Of Tanzania OR Tanzania OR Zambia OR Zimbabwe OR Rhodesia OR Africa Eastern OR Africa Southern OR Africa Western OR Africa Central

### 2.4 Screening of articles

The screening process of potential articles followed two steps: i) based on the abstract and title and ii) by full text. The first author screened the articles for eligibility. A second reviewer independently repeated 10% of the study screening at every stage through a systematic random selection of articles to minimize bias that may arise from human error. Rates of agreement between each of the independent reviewers and the first reviewer were assessed, and minor discrepancies resolved through consensus. Studies included fulfilled the following criteria highlighted in **Table 2**.

**Table 2:** Study selection criteria.

Inclusion criteria	Exclusion criteria
Population	
-Studies involving adult men and women aged ≥18 years (or whose mean/median age is within this age bracket) from SSA -Studies involving men or female alone or mixed sex (but with clear aggregations for males and females)	-Studies involving adult men and women outside SSA -Studies involving children, or adolescents -Studies with unspecified age range, mean or median age of participants -Studies which have not aggregated their results for males and females
Outcome	
-Studies on anxiety disorder or anxiety symptoms and associated factors -Studies on post-traumatic stress disorders and associated factors	-Studies on mental disorders other than anxiety and posttraumatic stress disorders or their symptoms -Studies where reported measurement scales do not evaluate anxiety or posttraumatic stress disorder or their symptoms
Study designs	
-Cross-sectional studies -Case-control studies -Cohort studies	-Review articles -Intervention studies -Case studies/reviews -Commentaries or editorials -Conference proceedings, symposia abstracts, or workshop publications -Qualitative studies -Books or book sections -Theses and dissertations

### 2.5 Data extraction

The first author (BM) extracted the following information from the included studies: a) *publication details* – the name of the first author and year of the publication; b) *details of the study* – country of origin, study design, study setting, data collection year; c) *study participant characteristics* – gender of study population (males, females, or mixed), sample size, age (mean, median, or range); d) *outcome and measures* – method of identification of CMD, prevalence of anxiety and PTSD, or their symptoms, measurement tool used, validity/reliability of outcome measure, the cut-off score used (for screening tools), factors associated with anxiety and PTSD or their symptoms (alongside the reported effect measure and precision estimate), and treatment of identified conditions. Only data that was aggregated by gender was extracted. For longitudinal studies, baseline values were extracted. Only significant factors identified in the multivariable analysis were recorded. A second reviewer (JM) independently extracted data from 20% of the included studies, and minor discrepancies discussed. The extracted data from the included studies is provided in **Supplementary File 4**.

### 2.6 Quality assessment

One author (BM) assessed the risk of bias of each of the included studies using Joanna Briggs Institute (JBI) tool. A second reviewer (JM) assessed the risk of bias for 20% of the included studies. The two authors settled discrepancies in quality rating. The JBI tool used is based on eight questions that generally assess the sampling methods (inclusion and exclusion criteria and description of the population of interest), measurement of the exposures and the outcomes (to establish outcome assessment validity and data collection process), methods put in place for generalizability of the findings, and the analysis done. The options for the questions were Yes (noted as high quality), No (low quality), Unclear (unclear risk) or Not Applicable. A cut-off score of less than 5 was considered indicative of low-quality studies and therefore excluded from the meta-analysis. This was selected in line with other reviews (35, 36) that adopted the same scores to ensure methodological rigor. Publication bias and heterogeneity was evaluated by visual inspection of funnel plots and statistical analysis using Egger’s test (37). The risk of bias assessment for the included studies is presented in **Supplementary file 1**.

### 2.7 Data analysis

We performed a meta-analysis of proportions with reported prevalence of anxiety and PTSD in the included studies stratified by sex. Heterogeneity between studies was quantified by the *I^2^* statistic. We considered *I^2^* values >50% to represent substantial heterogeneity. We used a random-effects model to derive the pooled estimates of anxiety and PTSD among the participants. The final prevalence estimates were reported as percentages with 95% confidence intervals. We also performed subgroup analysis for the different population types, the outcome assessment measure (whether screening or diagnostic tools used), and the different sub-Saharan regions. For studies that reported the correlates, we used a narrative synthesis of the factors associated with anxiety and PTSD with the odds ratios. Only the factors significantly associated with either anxiety or PTSD or their symptoms at *p <*0.05 in the multivariable analysis were considered and extracted as correlates. All the analysis was conducted in R software, version 4.5.0, using metafor package.

## 3 Results

### 3.1 Database search results

**Figure 1:**
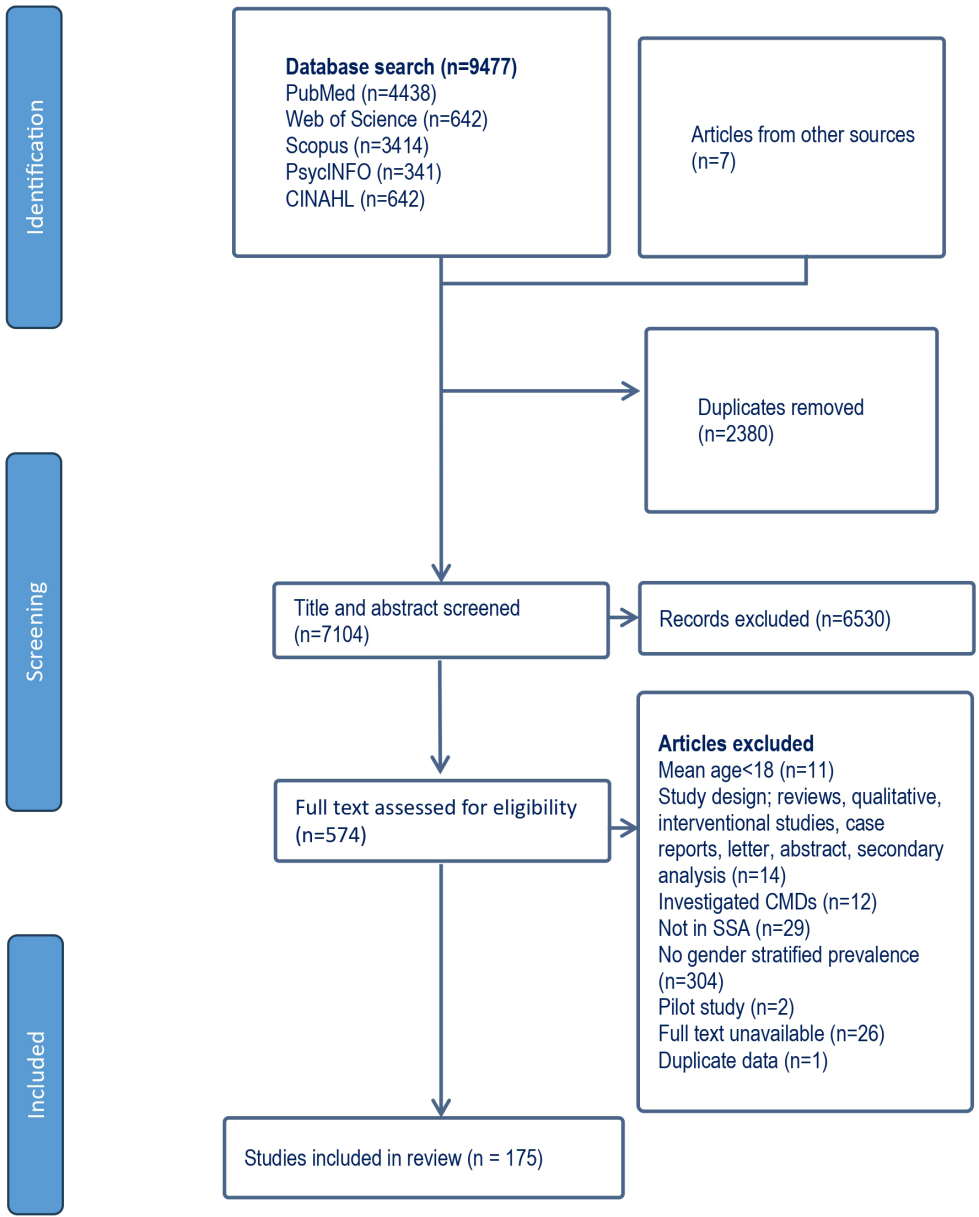
PRISMA flow chart for the study selection process.

**Figure 1** shows the PRISMA flowchart for the step-by-step systematic review process. 9477 articles were identified from five electronic databases, with additional 7 included from reference search of included studies. 7104 articles were included in the title and abstract screening phase after deduplication, and 574 later included in the full text screening. From this, 175 studies were included in the review. Three studies (38–40) were not included in the meta-analysis due to low bias score. The detailed search strategy and results have been provided in **Supplementary file 3**.

### 3.2 Overall characteristics of included studies

**Figure 2:**
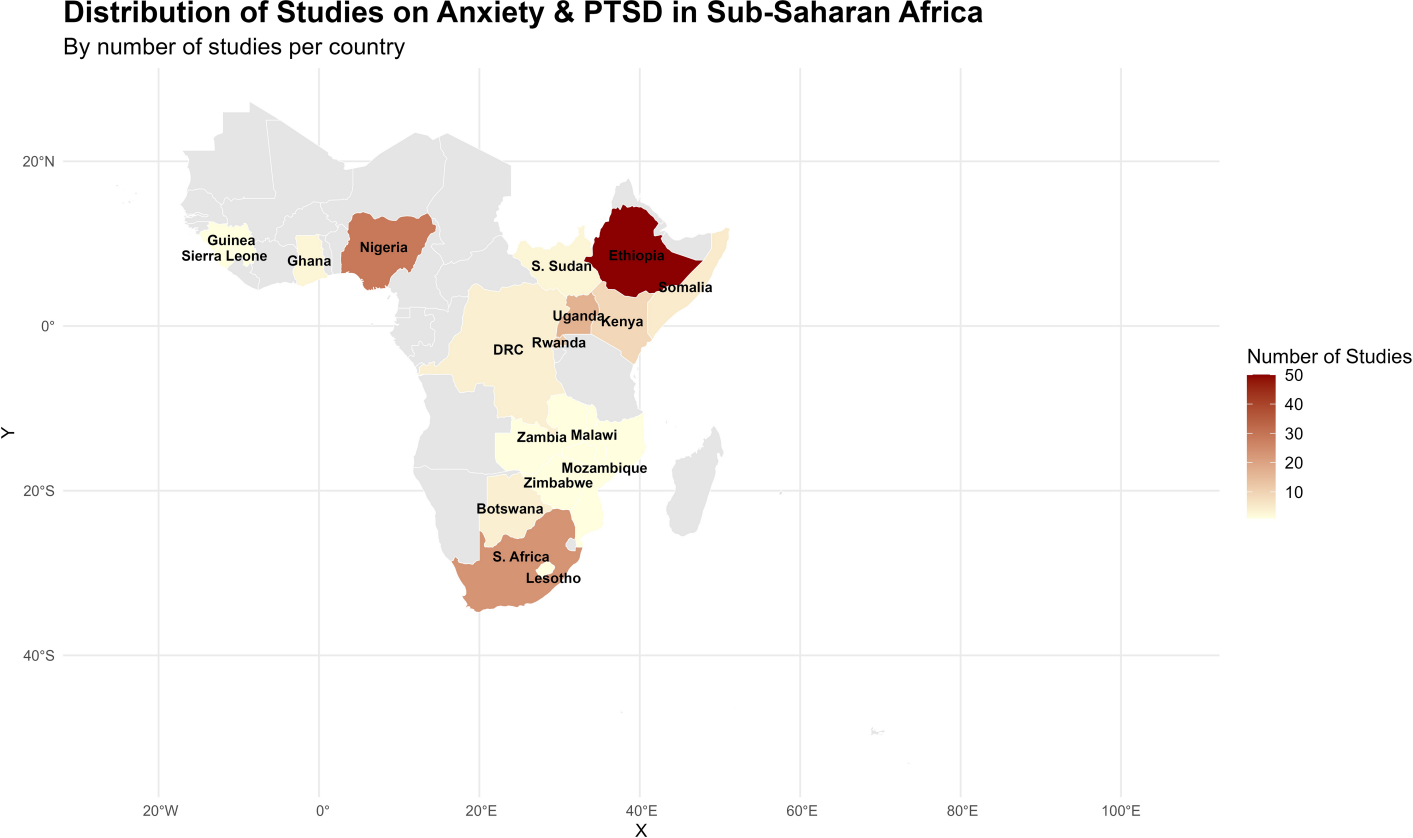
Distribution of anxiety and PTSD studies included in sub-Saharan Africa.

**Figure 3:**
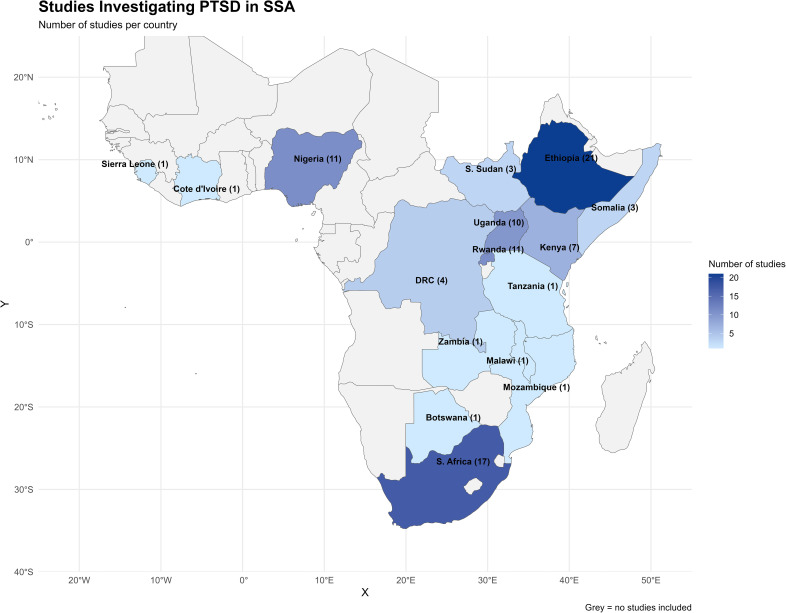
Distribution of PTSD studies in sub-Saharan Africa.

**Figure 4:**
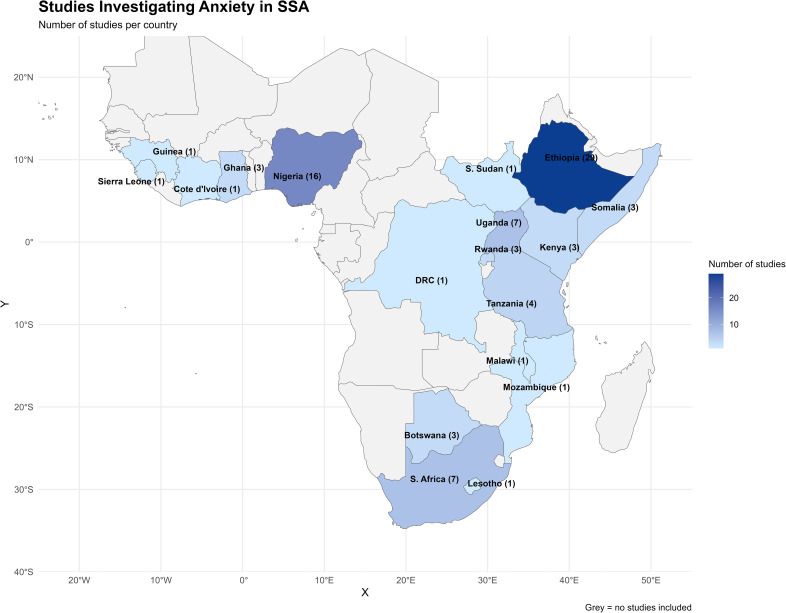
Distribution of anxiety studies in sub-Saharan Africa.

Overall, 75 studies reported anxiety outcomes only, 86 reported PTSD outcomes only, while 14 studies reported both anxiety and PTSD outcomes. The studies were conducted across 21 sub-Saharan African countries, majority (61%) in East African countries. **Table 3**, **Figures 2**–**4** highlight the geographical distribution of the studies.

**Table 3:** Geographical distribution of included studies by SSA regions.

Region	Studies, N (%)
East Africa	103 (60.6)
West Africa	33 (19.4)
Southern Africa	30 (17.6)
Central Africa	4 (2.4)

Some studies were multi-country, hence numbers add up to 170.

#### 3.2.1 Anxiety studies

60% [n=52] of the studies were conducted in Eastern Africa (41–92), 23% [n=20] in West Africa (93–112), 15% [n=13] in Southern Africa (113–125) and 1.2% [n=1] in Central Africa (126). All the studies had a cross-sectional study design. Majority of the studies (86.2%, n=75) used screening tools for assessment, and a few (13.8%, n=12) used diagnostic tools, shown in **Tables 4**, **5**.

**Table 4:** Utilization of anxiety screening tools by the included studies.

Screening tool	Number of studies (%)
Generalized Anxiety Disorder-7 Item Tool (GAD-7)	29 (33.7)
Hospital Anxiety and Depression Tool (HADS)	20 (23.2)
Depression, Anxiety and Stress Scale (DASS)	13 (15.1)
Hopkins Symptoms Checklist (HSCL-25)	4 (4.7)
Anxiety Rating Scale	1 (1.2)
Beck Anxiety Inventory (BAI)	1 (1.2)
Brief Symptom Inventory (BSI-18)	1 (1.2)
GAD-2	1 (1.2)
Generalized Health Questionnaire (GHQ-12)	1 (1.2)
Hamilton Anxiety Rating Scale (HAMA-A)	1 (1.2)
Patient Health Questionnaire (PHQ-4)	1 (1.2)
Social Anxiety Scale for Adolescents (SASA)	1 (1.2)
SCID-RV	1 (1.2)
Zung Self Rating Anxiety Scale	1 (1.2)

**Table 5:** Utilization of anxiety diagnostic tools by included studies.

Diagnostic tools	Number of studies (%)
Mini International Neuropsychiatric Interview (MINI)	6 (7.0)
Schedules for Clinical Assessment in Neuropsychiatry (SCAN)	2 (2.3)
Present State Examination (PSE 10)	1 (1.5)
Structured Clinical Interview for DSM Disorders (SCID)	1 (1.5)
Common Mental Disorder Questionnaire (CMDQ)	1 (1.5)

The studies investigated a wide range of population, including patients (n=16), PLWH (n=11), healthcare workers (n=12), perinatal women (n=10), war survivors (n=8), students (n=6), general population (n=5), youths (n=5), Ebola survivors (n=2), essential workers (n=2), caregivers (n=1), correctional officers (n=1), industrial park employees (n=1), older adults (n=1), people living with disabilities (n=1), prisoners (n=1), adults seeking HIV testing (n=1) and slum residents (n=1). The total female sample size was 30416, compared to 19362 males.

#### 3.2.2 PTSD studies

61.9% [n=60] of studies reporting PTSD outcomes were conducted in Eastern Africa (44, 53, 64, 69, 70, 79–81, 91, 127–175), 20.6% [n=20] in Southern Africa (118, 119, 121, 176–192), 13.4% [n=13] in West Africa (38, 95, 193–203), and 4.1% [n=4] in Central Africa. Based on the study design, 92.9% (n=91) were cross-sectional studies, 5 (5.1%) cohort studies, and 2 (2.0%) case-control studies. The distribution of PTSD studies using screening and diagnostic tools are presented in **Tables 6**, **7**. while Similar to anxiety studies, PTSD studies investigated different population groups, from war survivors (n=26), general population (n=18), healthcare workers (n=8), PLWH (n=8), road traffic accident survivors (n=6), perinatal women (n=4), security personnel (n=4), students (n=4), Ebola survivors (n=3), female sex workers (n=3), IPV victims (n=2), youths (n=2), caregivers (n=1) and survivors of different traumas (n=6). The total male sample size was 28805, while the female sample size was 40595.

**Table 6:** Utilization of PTSD screening tools by the included studies.

Screening tools	Number of studies (%)
PTSD Checklist (PCL)	38 (38.8)
Harvard Trauma Questionnaire (HTQ)	14 (14.3)
Impact of Event Scale (IES-R)	7 (7.1)
PC-PTSD-5	6 (6.1)
Breslau PTSD Screener	2 (2.0)
Post-Traumatic Diagnostic Scale (PDS)	2 (2.0)
PTSD Symptom Scale Report (PSS-SR)	2 (2.0)
Davidson Trauma Scale	1 (1.0)
National Stressful Events Survey PTSD Short Scale (NSESSS)	1 (1.0)
PTSD-8	1 (1.0)
Perinatal Post-traumatic Stress Disorder Questionnaire-11	1 (1.0)
S-PDS	1 (1.0)
Trauma Screening Questionnaire (TSQ)	1 (1.0)

**Table 7:** Utilization of PTSD diagnostic tools by the included studies.

Diagnostic tools	Number of studies (%)
MINI	15 (15.3)
Composite International Diagnostic Interview (CIDI)	2 (2.0)
SCIDI-RV	1 (1.0)
SPI	1 (1.0)
DSM Interview Guide	1 (1.0)

#### 3.2.3 Publication bias

**Figure 5:**
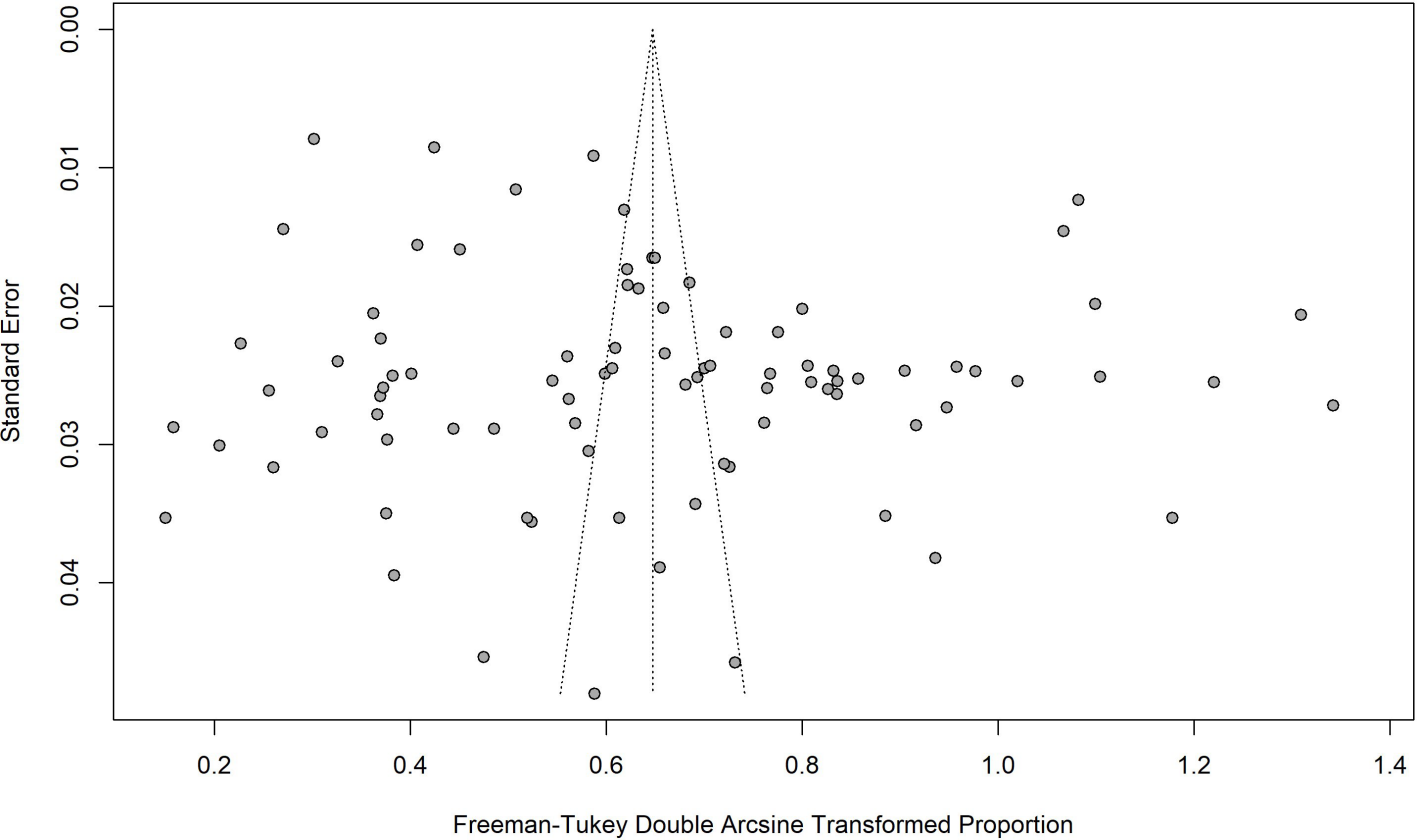
Funnel plot for anxiety studies.

**Figure 6:**
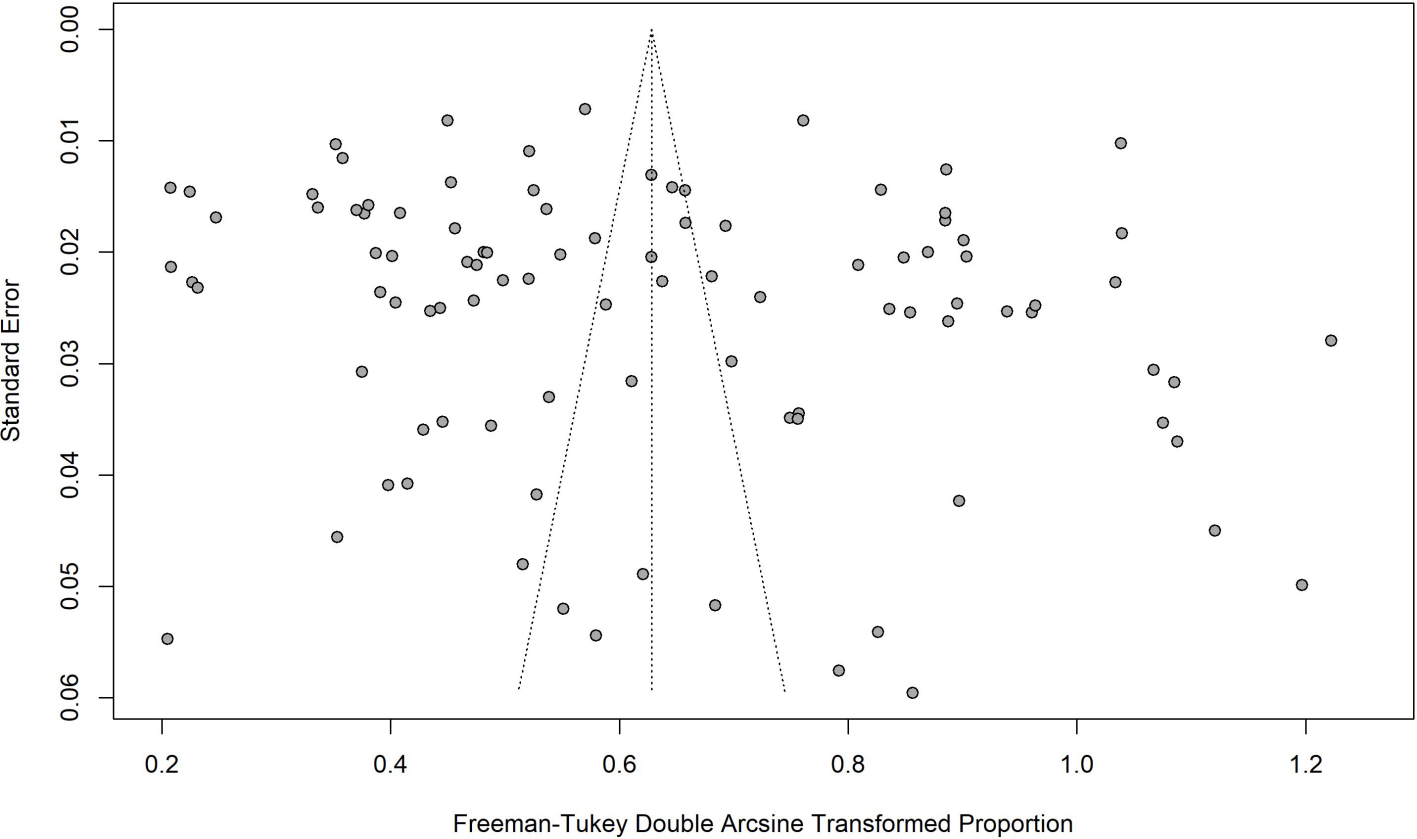
Funnel plot for PTSD studies.

Funnel plots shown in **Figures 5**, **6** highlight wide dispersions, with fairly asymmetrical distributions, suggesting publication or small study effects. The asymmetry was also supported by statistically significant Egger’s test for anxiety studies (t=2.09, df=85, p=0.0395). Egger’s test done for PTSD studies reported no statistically significant evidence of funnel plot asymmetry and publication bias (t = 1.36, df = 96, p = 0.178). The publication bias in this analysis should however be interpreted cautiously due to the high heterogeneity values between the studies.

### 3.3 Prevalence of anxiety

**Figure 7:**
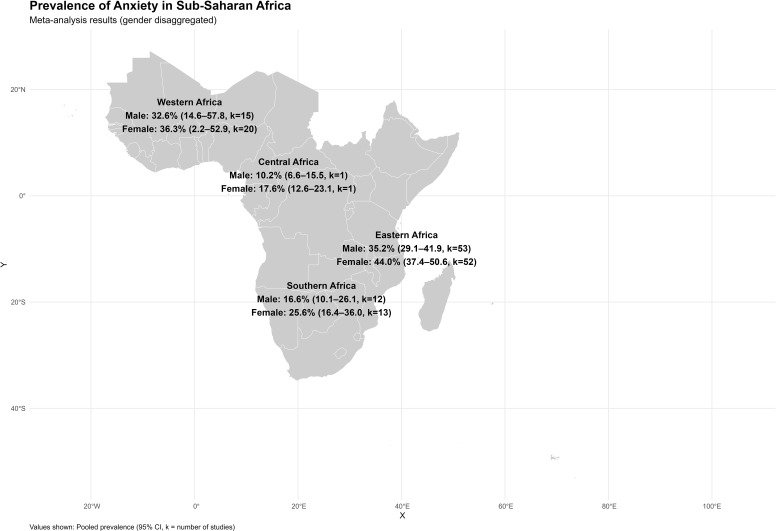
Gender-disaggregated anxiety prevalence in different sub-Saharan African regions.

**Figure 8:**
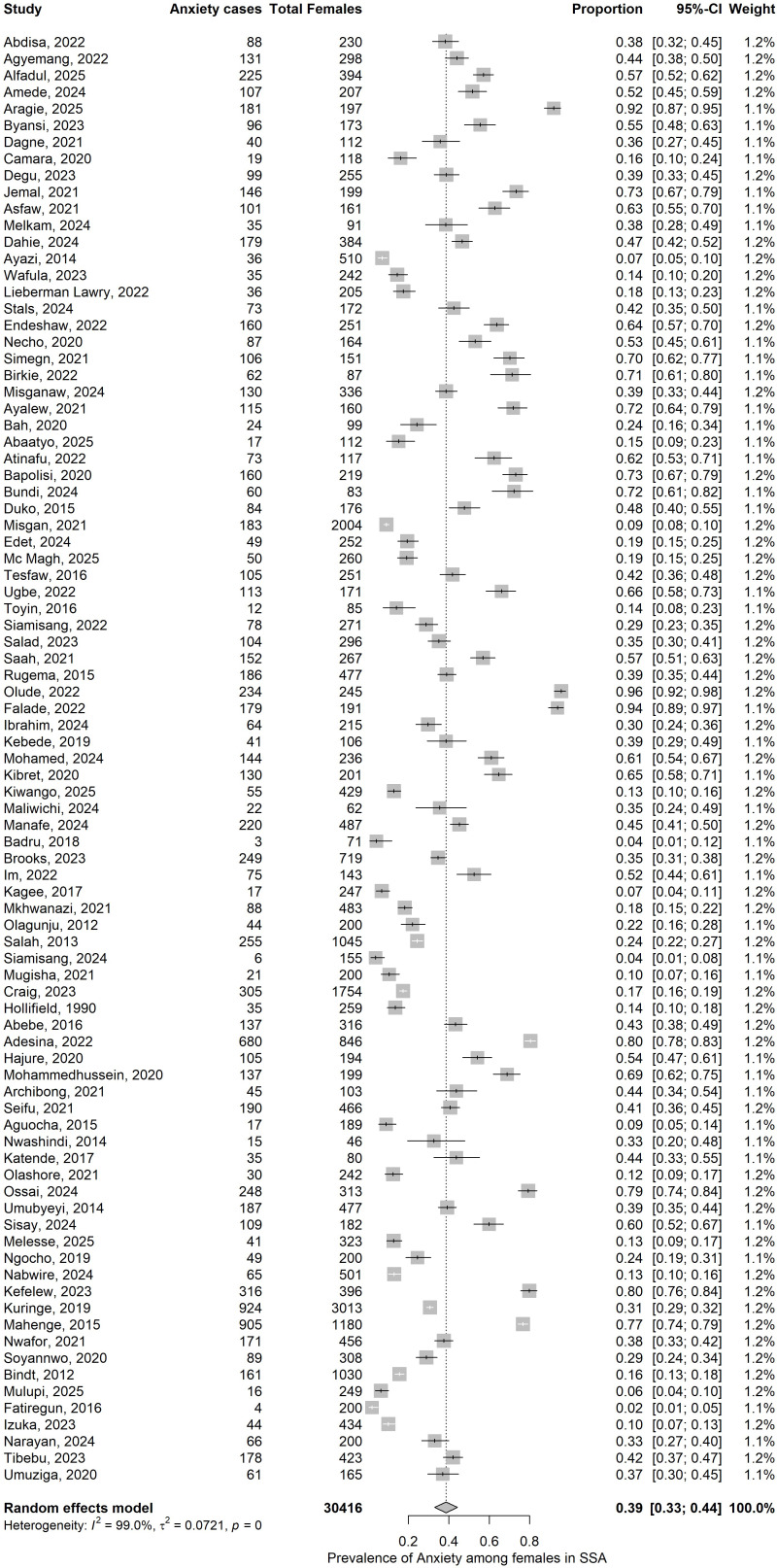
Forest plot for pooled prevalence of anxiety among females in SSA.

**Figure 9:**
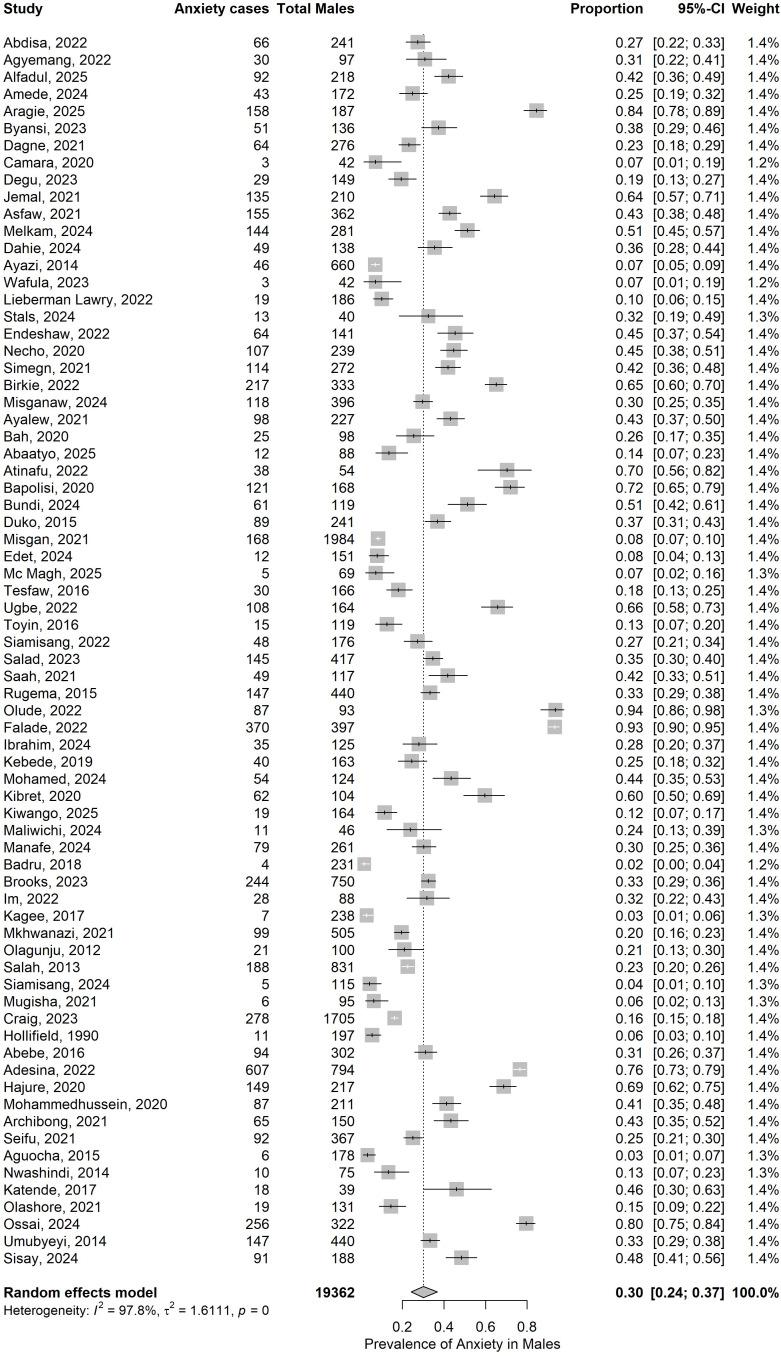
Forest plot for pooled prevalence of anxiety among males in SSA.

The overall prevalence of anxiety in sub-Saharan Africa was 36.35 (95% CI: 30.9%-41.9%, k=87), with significant difference noted in the regional prevalence. Eastern Africa had the highest overall pooled prevalence, 41.6% (95% CI: 35.4%-47.9%, k=52), followed by West Africa, 34.0% (95% CI: 19.1%-50.7%, k=20). Southern Africa had the lowest pooled prevalence, 23.7% (95% CI: 15.4%-33.3%, k=13). Only one study from Central Africa was included, hence no analysis was done for the region. Significant differences were noted in the male and female prevalence in the different sub-Saharan African regions, shown in **Figure 7**. Overall, women had 39% pooled prevalence, while men had 30%, shown in **Figures 8**, **9**.

**Figure 10:**
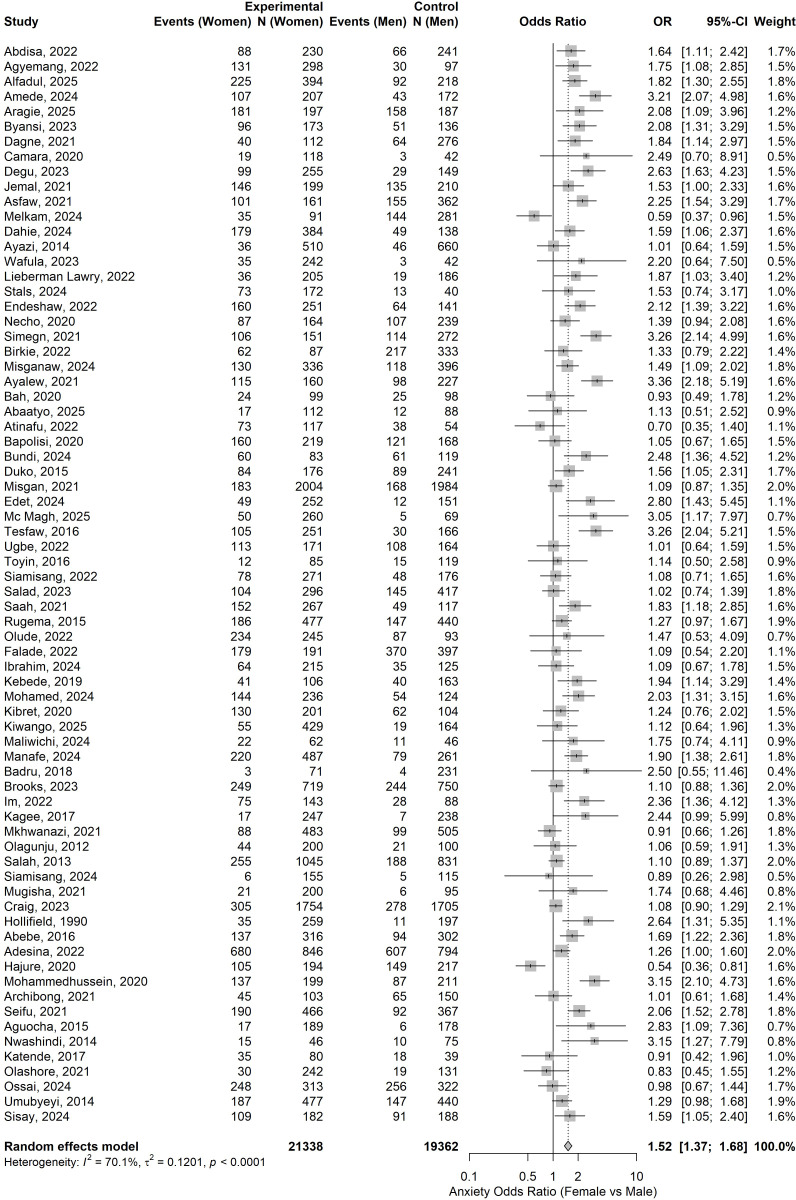
Female: male odds ratio for anxiety studies.

**Figure 10** is a forest plot of 72 studies comparing anxiety prevalence between men and women, showing significantly higher odds of anxiety (OR = 1.52, 95% CI: 1.37-1.68, p <0.0001) in women, although heterogeneity across studies was substantial (I^2^ = 70.1%).

Significantly higher odds were also noted in the regional analysis, with slightly high odds in East Africa: (OR = 1.56, 95% CI: 1.36-1.78, p<0.0001) compared to southern Africa (OR = 1.44, 95% CI: 1.14-1.81, p<0.0019) and West Africa: (OR = 1.41, 95% CI: 1.16-1.72, p =0.0005). One study from Central Africa showed higher odds ratio in females (OR = 1.87, 95% CI: 1.03-3.40, p = 0.0390).

The pooled prevalence for the total population in the studies that used screening tools was 38.7% (95% CI: 33.0%-44.5%, k=75). Females had a pooled prevalence of 41.1% (95% CI: 35.2%-47.1%, k=75), compared to males’ 34.0% (95% CI: 27.9%-40.4%, k=61).For those that used diagnostic tools for assessment, estimate prevalence was 22.6% (95% CI: 9.1%-39.8%, k=12), among 24.2% (95% CI: 10.6%-41.2%, k=12) among women and 23.4% (95% CI: 8.9%-42.1%, k=11) for men.

#### 3.3.1 Anxiety subgroup analysis by study population

Subgroup analysis for the study population in females showed higher prevalence among war survivors (49.1%, 95% CI: 35.7%–62.5%, k=8), students (45.0%, 95% CI: 22.6%–68.5%, k=6) and health care professionals (44.5%, 95% CI: 26.6%–63.1%, k=12). Prevalence among the general population was 29.4% (95% CI: 2.4%–70.0%, k=5), patients 37.3% (95% CI: 27.5%–47.6%, k=26), and youths 30.4% (95% CI: 20.2%–41.7%, k=5). The lowest prevalence was seen among perinatal women (25.0%, 95% CI: 10.4%–43.4%, k=9).

In male subgroup analysis by population types, pooled prevalence ranged from 24.8% (95% CI: 1.0%–65.4%; k = 5) in the general population to 39.3% (95% CI: 24.4%–55.4%; k = 8) among war survivors. Studies with healthcare workers had 34.3% (95% CI: 18.9%–51.6%; k = 12) prevalence, youths: 34.1% (95% CI: 4.0%–74.8%; k = 3), students: 31.6% (95% CI: 19.0%–45.9%; k = 6), and patients: 29.0% (95% CI: 20.0%–39.0%; k = 24). The differences in the study populations for both males and females were, however, not statistically significant.

### 3.4 Prevalence of PTSD

**Figure 11:**
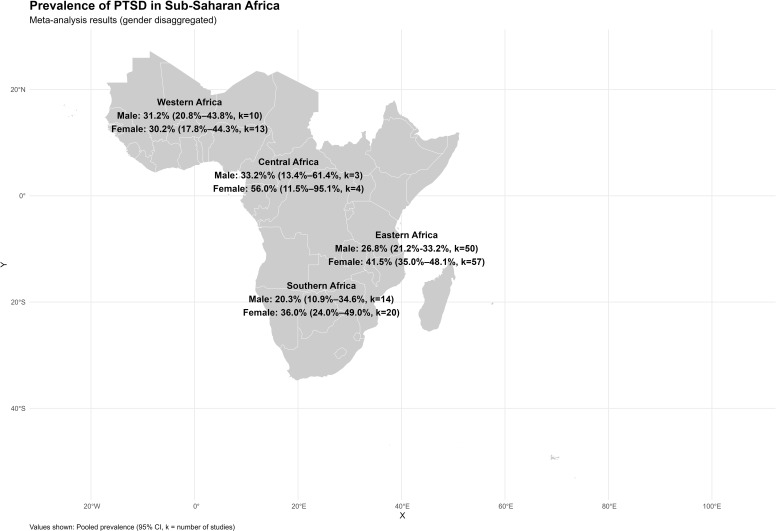
Gender-disaggregated PTSD prevalence in different sub-Saharan African regions.

Pooled PTSD prevalence for the total population in the included studies was 34.5% (95% CI: 29.8%-39.4%, k=98). Subgroup analysis by region showed wide prevalence ranges, though not statistically significant (p: 0.7017). There was a relatively high prevalence in Central Africa from the 4 studies included: 52.4% (95% CI: 7.7%-94.7%, k=4), and comparatively similar prevalence in Eastern Africa: 34.6% (95% CI: 28.7%-40.6%, k=60), Southern Africa: 33.8% (95% CI: 22.1%-46.5%, k=20), and West Africa: 31.3% (95% CI: 17.4%-47.2%, k=13). Using a random effects model, the pooled prevalence of PTSD among women was 39.1% (95% CI: 34.0%–44.4%, k=95), relatively higher compared to the prevalence in males: 26.24% (95% CI: 21.76%–31.28%, k=78). **Figure 11** shows the gender-stratified pooled prevalence of PTSD in different SSA regions. The difference in regional prevalence estimates for both males and females was, however, not statistically significant.

**Figure 12:**
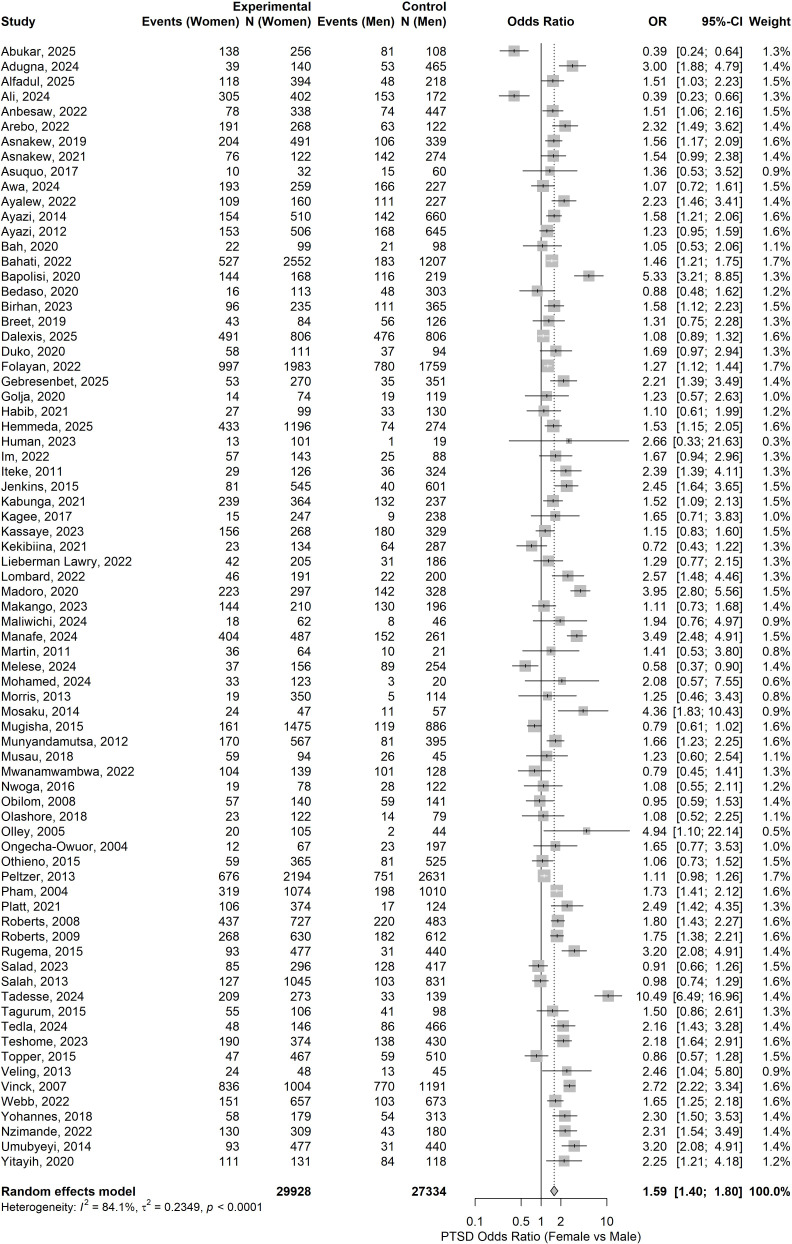
Female: male odds ratio for PTSD studies.

Studies with both male and female participants showed significantly higher odds of PTSD among the females (OR = 1.59, 95% CI: 1.40-1.80, p <0.0001), with significant heterogeneity between the studies (I^2^ = 84.1%). **Figure 12** shows the forest plot for the studies included. Additional forest plots are provided in **Supplementary File 2**. In the regional analysis, higher odds of PTSD in women were consistently noted in most regions. In Eastern Africa, 47 studies showed significantly higher odds of PTSD in women (OR = 1.64, 95% CI: 1.39-1.94, p <0.0001), with significant heterogeneity in the studies (I^2^ = 86.8%). 10 studies from West Africa showed OR = 1.33, 95% CI: 1.08-1.62, p= 0.0060. 14 studies from Southern Africa showed slightly higher odds (OR = 1.58, 95% CI: 1.17-2.12, p= 0.0024). Central Africa showed insignificantly higher odds from the 3 studies included (OR = 1.25, 95% CI: 0.90-1.74, p=0.1762).

Studies that used screening tools had a pooled prevalence of 38.7% (95% CI: 33.3%-44.2%, k=77), with a higher pooled prevalence in females, 43.1% (95% CI: 37.5%-48.8%, k=75), compared to males, 31.5% (95% CI: 26.3%-36.9%, k=58).

Those using diagnostic assessment tools had 18.0% (95% CI: 11.8%-25.2%, k=20) estimate, 25.0% (95% CI: 15.0%-36.4%, k=19) among women. and 18.5% (95% CI: 9.9%-29.0%, k=19) in men.

#### 3.4.1 PTSD sub-group analysis by study population

Subgroup analysis by study population among females showed significant differences in PTSD prevalence (Q = 73.72, df = 7, p < 0.0001). The highest pooled prevalence was observed among war survivors (58.4%, 95% CI: 49.3%–67.3%; k = 24), followed by healthcare professionals (45.3%, 95% CI: 22.2%–69.5%; k = 8) and patients (39.3%, 95% CI: 21.0%–59.3%; k = 9). Lower pooled prevalence estimates were observed among students (37.3%, 95% CI: 2.1%–84.2%; k = 4), the general population (32.8%, 95% CI: 23.9%–42.4%; k = 18), and RTA survivors (27.0%, 95% CI: 14.7%–41.4%; k = 6). The lowest pooled prevalence was found among perinatal women (13.6%, 95% CI: 0.9%–37.3%; k = 4) and youths (21.4%, 95% CI: 4.4%–46.4%; k = 2). In males, subgroup analysis by study population revealed substantial variability in the prevalence estimates (Q = 43.84, df = 6, p < 0.0001). War survivors had the highest prevalence (46.9%, 95% CI: 35.9% to 57.9%, k=20), followed by health care professionals (33.8%, 95% CI: 14.4% to 56.3%, k=8), patients (25.6%, 95% CI: 9.2% to 46.5%, k=7), and the general population (23.7%, 95% CI: 16.5% to 31.8%, k=17). Lower prevalence estimates were found among road traffic accident (RTA) survivors (15.3%, 95% CI: 10.8% to 20.3%, k=6), students (19.4%, 95% CI: 9.8% to 31.3%, k=3), and youths (10.9%, 95% CI: 0.0% to 85.3%, k=2).

### 3.5 Factors associated with anxiety

A few (15.9%, n=14) of the included studies reported gender-stratified correlates, highlighted in **Table 8**. Generally, the correlates noted were biological, occupational, social and psychological, educational, obstetrics/health-related, socioeconomic, and early life and developmental factors. In males, socioeconomic factors and urban residence were majorly reported from two studies (53, 122). Adverse childhood experiences was the only early life correlate reported in males (122). In educational factors, men with no formal education reported significantly lower anxiety prevalence in one of the studies (93).

**Table 8:** Factors associated with anxiety.

Study	Country	Study population	Male-associated factors (AOR)	Female associated factors (AOR)
Agyemang, 2022 (93)	Ghana	PLWH	No formal education (0.18)	Non tertiary education level (0.32) No formal education (0.55)
Melesse, 2025 (47)	Ethiopia	Perinatal women		Single marital status (2.29) Unplanned pregnancy (1.99)
Ayazi, 2014 (53)	South Sudan	General population	Urban residence (2.88) Moderately disadvantaged socioeconomic position (4.69) Severely disadvantaged socioeconomic position (4.85)	Severely disadvantaged socioeconomic status (3.53)
Ngocho, 2019 (55)	Tanzania	PLWH		Marital status- single (3.6) Ever experienced violence (2.3) HIV shame (1.1)
Nabwire, 2024 (71)	Uganda	Perinatal women		Monthly income =<200000 (2.75), [ref >200000] Strained marital relationship (2.52) HTN complication in past pregnancy (4.17)
Kefelew, 2023 (75)	Ethiopia	Industrial Park employees		Marital status- single (1.99) Poor level of social support (3.78) Overtime work (2.31) 3–4 years work experience [ref >5 years] (4.71) Fear of job loss (1.72)
Kuringe, 2019 (78)	Tanzania	Youths		Incomplete or complete secondary education (1.36) Living with husband (1.23) HIV positive serostatus (1.54) Violence from sexual partner in last six months (1.63) Sex work in the last six months (1.31)
Mahenge, 2015 (80)	Tanzania	Perinatal women		Employment status- self employed (1.56) - [ref - employed]. Age 35-43 (0.60)- [ref 30-34] Partners education- secondary (2.08) [ref partner primary education] Partners education-college (2.25) [ref partner primary education]. <1 year relationship duration (0.56) [ref >1 year duration]
Mkhwanazi, 2021 (122)	South Africa	Youths	Poverty- stealing in last month because of hunger (1.80) Adverse childhood experiences (1.04) Experience three of more adverse events in the lifetime (2.20)	Poverty- stealing in the past month because of hunger (2.03) Stress because of lack of work (1.12) Violence- Past year IPV (1.83) Experience three of more adverse events in the lifetime (2.17)
Nwafor, 2021 (103)	Nigeria	Perinatal women		38–45 years (2.2) [ref 18-27] Parity >=5 (4.1) [ref 0 parity] Urban residence (2.6) Tertiary education level (3.8) [ref no formal education] Occupation- trader (4.2) [ref housewife]
Soyannwo, 2020 (105)	Nigeria	Youths		Gynaecologic conditions (2.7)
Mulupi, 2025 (83)	Kenya	Perinatal women		Currently married (β -1.87) Tertiary education (β 1.18) [ref primary and no education] 4 => pregnancies (β 2.50) [ref nulliparous]Little pain interference with normal work in past 4 weeks (β 3.29) [ref No pain] Moderate to extreme pain interference with normal work in 4 weeks (β 2.23) Consulted health professional for not feeling well, (β 1.14) [ref no consultation]
Tibebu, 2023 (89)	Ethiopia	PLWH		Unplanned pregnancy (1.09) Didn’t discuss HIV with sexual partner (3.21)
Umuziga, 2020 (90)	Rwanda	Perinatal women		Constant stressful life events (2.70)

Among the females, a wide range of factors were reported.

Biological factors: HIV-seropositive women (78), those who had physical pain during normal work (83), and older adults [>35 years] (80, 103) were significantly associated with higher anxiety prevalence in females.

Occupational factors: Women who worked overtime, had fewer years of working experience, had a fear of job loss (75), were self-employed (80, 103), those who had stress because of work (122) and those who were sex workers (78) had significantly higher odds of anxiety.

Social and psychological factors: One study (71) reported women with strained marital relationships to have higher odds of anxiety, with another noting women with less than one year in a relationship to have significantly lower odds of anxiety (80). Intimate partner violence was another significant factor noted (55, 78, 122). HIV-related stigma/shame (55) and women who did not discuss HIV with their sexual partner (89) also had higher odds of having anxiety. Social support was a notable factor, with women who had constant stressful life events (90) and poor social support (75) having significantly higher anxiety odds.

Health-related/obstetrics factors: Pregnancy and gynecological complications (71, 105), unplanned pregnancy (47, 89), and multiparity (83, 103) were noted obstetric factors.

Sociodemographic factors: Urban residence (103), disadvantaged socioeconomic position (53), low monthly income (71), and partner’s education level (80) were reported across the studies. Most studies reported similar results related to marital status. Several studies (47, 55, 75, 78) reported single and unmarried women to have significantly higher anxiety prevalence, with lower coefficients for ladies in marriage (83). No specific group of women in terms of the education level showed higher dominance in developing anxiety. Those with no formal education (93), completed secondary education (78), and tertiary education (83, 103) showed significantly higher odds of anxiety.

Early life and developmental factors: Adverse Childhood Experiences (122) were noted among females, similar to males.

### 3.6 Factors associated with PTSD

Studies that reported PTSD correlates are highlighted in **Table 9**. Most of the significant factors reported were trauma-related, and majorly among the females. The factors majorly fell in the following themes:

**Table 9:** Factors associated with PTSD.

Study	Country	Study population	Male correlates (AOR/β)	Female correlates (AOR)
Alemu, 2024 (129)	Ethiopia	Perinatal women		Primipara (2.26) No ANC follow-up with recent baby (2.48) CS delivery (2.86) Instrumental delivery (3.06) Maternal morbidity during pregnancy, delivery or PP period (2.94) IPV during PP period (7.43)
Ayazi, 2014 (53)	South Sudan	General population	Moderately disadvantaged socioeconomic status (2.35) Trauma exposure during war (1.28) Trauma exposure after peace agreement (1.08)	Urban residence (3.46) Moderately disadvantaged socioeconomic status (5.26) Severely disadvantaged socioeconomic status (1.89) Trauma exposure during war (1.46) Trauma exposure after peace agreement (1.12)
Baguma, 2024 (138)	Uganda	Security personnel	Age 35-44 (β 0.72) [ref 45>] Married (β 1.21)	
Beksinska, 2021 (141)	Kenya	Female sex workers		Adverse family/household experience (2.36) Experienced sexual/physical violence in childhood (2.03) Street homeless as a child (1.79) Higher number of ACEs;9-12 (7.19) [ref =<4] Social support (0.59) Has 3+ child (0.31) [ref None] Nonconsensual first sex (2.75) Recent anal sex (11.7) chlamydia infection (2.46) Had an abortion/still birth (1.60) Recent sexual/physical violence from IPs (1.68) Recent sexual/physical violence from non IP (1.68) Alcohol use (4.49)
Dossa, 2014 (204)	DRC	War survivors		CRSV and fistula (β 0.466) [ref No SV/fistula) CRSV and no Chronic Pain (β 0.18) [ref No SV/CP] CRSV and Constant Pain (β 0.405) [ref No SV/CP]
Gupta, 2014 (196)	Cote d’Ivoire	IPV victims		Remote IPV (2.6) Primary, secondary or higher education (0.6) Traditional religion (4.0)
Mahenge, 2015 (80)	Tanzania	Perinatal women		Partners age 35-58 (2.80) [ref 30-34] Marital status- not married/cohabiting (2.71) [ref Married] <1 year relationship duration (0.27) [ref >1]
Mukabana, 2023 (160)	Kenya	Caregivers		Previous history of preterm delivery (0.09) CS delivery (11.1) Fair housing condition (16.5) [ref good housing condition]
Nzimande, 2022 (191)	South Africa	General population	Gets enough emotional support from family members (0.26)	Social isolation (1.15) Gets enough emotional support from family members (0.27)

Early life traumatic experiences: Adverse childhood experiences, both physical and sexual, were noted among the females from one study (141).

War-related traumatic events: Trauma exposure during war and after peace agreements (53) was noted in both males and females, with females who had conflict-related sexual violence (CRSV) with physical complications (204) also reporting higher odds.

Social/interpersonal/psychological factors: In both males and females, social support was seen as a protective factor, with those having emotional support and social interaction showing significantly lower PTSD prevalence (141, 191). Past and recent intimate and non-intimate partner violence was also reported among females (42, 141, 196).

Obstetrics/health-related factors: Primiparous women (205), those with a history of abnormal delivery, cesarean delivery (160, 205), instrument delivery, maternal morbidity during pregnancy, delivery, or postpartum (205), and a history of abortion/stillbirth (141) were highly associated with PTSD among women. In one study (160), those with a history of preterm delivery showed lower odds of developing PTSD.

Socioeconomic/demographic factors: Disadvantaged socioeconomic status was a significant factor reported in both males and females (53). Urban residence (53) and housing conditions (160) were factors reported only in females. A study investigating defense force soldiers (138) reported married men to have a significantly higher prevalence of PTSD. Perinatal women who were not married had higher PTSD prevalence (80).

## 4 Discussion

### 4.1 Summary of key findings

This review aimed to synthesize gender-stratified prevalence and correlates of anxiety and PTSD among adults in SSA. Research on anxiety and PTSD in SSA is growing, evidenced by the high number of studies included. However, the quantitative evidence is focused on a small number of countries in Eastern, Western, and Southern Africa regions. Only four studies from Central Africa were included in the review. Exclusion of non-English studies limited inclusion of potential relevant studies published from the French speaking countries, and the generalizability of the existing evidence in the specific region. The studies also mainly focused on middle-aged adults, with only one (50) involving older adults above 65 years of age. The prevalence of anxiety and PTSD was generally high in sub–Saharan Africa, regardless of gender. For anxiety, females had a higher pooled prevalence (39%) compared to men (30%). PTSD, similarly, recorded a higher prevalence in women (39%) compared to men (26%). Females had higher odds of anxiety (1.52) and PTSD (1.59). There was a limitation in reporting gender-specific correlates, highlighting a gap in understanding the specific factors and research in SSA. The studies predominantly reported factors contributing to anxiety and PTSD among women, with limited evidence among men. For men, demographic and traumatic experiences were noted to contribute to both anxiety and PTSD. Women reported more traumatic, demographic, psychosocial, and obstetric-related factors.

### 4.2 Prevalence of anxiety and PTSD

Higher prevalence patterns of anxiety and PTSD have been shown among females in most of the studies that reported gender-stratified prevalence. The significantly higher prevalence of anxiety and PTSD in the different regions is consistent with global patterns noted. A global burden of disease (GBD) review on anxiety (206) also noted this across the lifetime in different regions. Overall, the GBD review noted a male-to-female ratio of 0.46, which was higher than the SSA findings in this review. The gender difference patterns persisted regionally, though there were variations in the magnitude of the prevalence identified. Regional differences in the prevalence of PTSD were, however, not significant. Similar gender disparities were noted in PTSD findings, though not uniform in the different regions and study populations. Differences in these measures seen may be brought about by varying experiences, resilience and life events at different points. A recent meta-analysis study on gender differences in mental health disorders among people exposed to natural hazards reported women to be 80% more likely to have PTSD compared to men (207). This was higher than the findings from this review, where war-affected females had 56% higher odds of being affected by PTSD. In the regional comparison, higher prevalence in females was recorded in all regions except in West Africa, where 28% female PTSD prevalence was recorded, compared to males’ 31%. Eastern Africa region had the highest prevalence of anxiety, consistent with previous findings (206). Different exposures to life experiences and stressors have been highly noted in Eastern Africa and Western Africa regions (12), highly known for high cases of displacement, and more affected by traumatic experiences. Significant differences were recorded in the male and female PTSD prevalence, contrasting previous findings by Ng et al. (32) that reported an insignificant difference. There was a notable wide prevalence distribution in both anxiety and PTSD in different regions, highly likely brought about by the diversity in the study populations and assessment methods used.

### 4.3 Correlates of anxiety and PTSD

In the studies, majority of the correlates noted were focused on women. Marital status identified majorly among women noted those in marriage had relatively lower odds of anxiety and PTSD. Interpersonal relationships, and specifically marriage, have been noted by other studies to have contrasting influence on mental health disorders. For instance, a systematic review focusing on factors contributing to gender differences noted interpersonal relationships to have a dual effect on women’s anxiety prevalence, noting that they are the main source of anxiety, and, at the same time, have a protective role linked to social network support and expressiveness (208). Traumatic experiences were the most widely noted factors in both anxiety and PTSD. Childhood and adulthood experiences, noted among females, were mostly sexually related, with some physical complications. Child emotional abuse and neglect have been reported to have a lasting impact on mental health in adulthood in previous literature, leading to suicidal ideation, anxiety, depression, and other psychological symptoms (209). The findings emphasize the impact of early trauma in shaping psychological and emotional response and development. Traumatic experiences have also been reported in other reviews to highly contribute to females’ higher anxiety, linked to rumination and anxiety sensitivity (208).

Concerning trauma experience and effect on PTSD, differences in the overall number of traumatic experiences have been noted in other studies, and despite men reporting a generally high number of traumatic events, women show significantly higher prevalence of lifetime PTSD (210). For example, both men and women experience gender-based violence, but women have been reported to have a threefold higher prevalence of PTSD as a result. A study done in a war-affected population in Lebanon investigating the association of trauma type and gender difference in PTSD noted some agreeing factors (211). Women who had a high number of adverse life events, physical health problems and those who had witnessed traumatic events had an increased risk of PTSD. Both males and females experience domestic violence, though the association was much higher among females. A few protective factors were highlighted in some of the studies, including social and emotional support, and younger ages.

Majority of the identified factors in the studies are easily modifiable, given that most of them stem from environmental exposures and social interactions rather than fixed characteristics, highlighting potential for targeted interventions. The findings from this study emphasize the need to note specific factors relating to anxiety and PTSD in males and females. Identifying the interrelation of biological, cultural, and environmental factors may help explain the differences and inform gender-specific mental health interventions. A closer look into the gender roles and understanding of different societies, i.e. broadly, egalitarian and hierarchical societies would help explain some of the differences in gender roles and how they contribute to the differences in mental health disorders.

### 4.4 Implications of the study

Mental health research in SSA has limited presentation of gender-specific correlates, which has hindered understanding, and generalization of the few identified factors across different populations. Future studies should incorporate gender-sensitive frameworks and tools to better identify and analyze factors specific to both men and women. Furthermore, research on cultural influence on sex differences in mental health should be considered to better understand contextual differences and gender relationships that bring about noted differences. Prioritizing the inclusion of the elderly population should also be considered. Targeted efforts should aim to address the more pronounced factors contributing to these mental health conditions, including violence, traumatic-related factors, and sociodemographic vulnerabilities. There is a need to design and adapt gender-sensitive preventive and curative mental health interventions. Focused psychosocial support, trauma-informed counselling, and mental health programs should be integrated into clinical settings and address the different sex needs.

### 4.5 Study strengths and limitations

This review has a wide evidence base, synthesizing 175 studies across different populations and regions, that tries to give a true picture of the mental health burden in SSA. Considering we only included significant factors in the multivariable analysis, this reduced the risk of over interpreting unadjusted correlates. This, however, might also have left out some significant but underreported correlates with the two outcomes. Most of the studies did not report gender-disaggregated correlates, limiting a comprehensive synthesis across different populations. The differences in the study populations, locations and contexts, that may have influenced the correlates being looked into made it difficult to clearly come up with adjusted models for comparison. The correlates were more reported among females, making it difficult to draw conclusions about gender differences in the factors affecting anxiety and PTSD. Central Africa region was not well represented, likely because of the search restriction to English language studies that may have introduced regional bias by under-representing francophone countries in the region. This made it difficult to generalize and compare the findings to other regions. The cross-sectional nature of most of the studies included could not allow longitudinal comparison of the factors contributing to the differences at different points in time. In addition, the studies leaned more towards using screening tools that have been previously shown to lead to both overestimation and underestimation, depending on the cultural and regional background of the population being screened (212). The false positive rates with the screening tools high likely exaggerate the true prevalence of the disorders. High heterogeneity across the studies and sub group analysis indicating considerable variability also limits the interpretation and generalizability of the found estimates to the larger SSA region and study populations. The found pooled measures should therefore be interpreted not as exact estimates for the regions but rather broad summaries.

## 5 Conclusion

Overall, the prevalence of anxiety and PTSD is high in SSA, and with all genders. Gender sensitive anxiety and PTSD research in SSA is growing, but has barely gone beyond the numbers to identify the specific factors and contextual differences that contribute to the prevalence noted, especially among men. Addressing this research gap is crucial in developing gender and culturally sensitive interventions in different SSA regions. Future studies should aim at investigating multifaceted factors, both socially constructed and biological differences that influence mental health disorders.

## Data Availability

The original contributions presented in the study are included in the article/**Supplementary Material**. Further inquiries can be directed to the corresponding author.

## Supplementary material

Supplementary File 1 Risk of bias assessment.

Supplementary File 2 Forest plots for anxiety and PTSD prevalence in SSA.

Supplementary File 3 Search strategy and results.
