# Supplementary material for: Gender differences in the prevalence of anxiety and post-traumatic stress disorder and associated factors among adults in sub-Saharan Africa: a systematic review and meta-analysis
Source: Front Psychiatry. 2026 Jul 23;17:1817602. doi: 10.3389/fpsyt.2026.1817602 (PMC13444377; doi:10.3389/fpsyt.2026.1817602)
Supplement: Supplementary file 1 [file Supplementaryfile2.docx]

**Supplementary Figure 1:** Prevalence of anxiety in sub-Saharan Africa Forest plot.

**Supplementary Figure 2:** Prevalence of PTSD in sub-Saharan Africa Forest plot

**Supplementary figure 3:** PTSD prevalence among females in sub-Saharan Africa

**Supplementary figure 4:** PTSD prevalence among males in sub-Saharan Africa
