# Supplementary material for: Gender differences in the prevalence of anxiety and post-traumatic stress disorder and associated factors among adults in sub-Saharan Africa: a systematic review and meta-analysis
Source: Front Psychiatry. 2026 Jul 23;17:1817602. doi: 10.3389/fpsyt.2026.1817602 (PMC13444377; doi:10.3389/fpsyt.2026.1817602)
Supplement: Supplementary file 2 [file Supplementaryfile3.docx]

| **SEARCH STRATEGY DATE; 16/5/25** | | |
| --- | --- | --- |
| **DATABASE** | **STRING** | **RESULTS** |
| **PUBMED** | (("Anxiety"[Title/Abstract] OR "anxiety disorders"[MeSH Terms] OR "generalized anxiety disorder"[MeSH Terms] OR "anxi*"[Title/Abstract] OR "anxiety symptoms"[Title/Abstract] OR "symptoms of anxiety"[Title/Abstract] OR "GAD"[Title/Abstract] OR "posttraumatic stress disorder"[Title/Abstract] OR "stress disorders, post traumatic"[MeSH Terms] OR "PTSD"[Title/Abstract] OR "traumatic stress"[Title/Abstract] OR "post traumatic stress"[Title/Abstract] OR "trauma"[Title/Abstract] OR "psychological trauma"[Title/Abstract] OR "psychological trauma"[MeSH Terms] OR "stress disorders"[Title/Abstract]) AND ("adult"[MeSH Terms] OR "old age"[Title/Abstract] OR "elder*"[Title/Abstract]) AND ("africa south of the sahara"[MeSH Terms] OR "africa south of the sahara"[Title/Abstract] OR "sub saharan africa"[Title/Abstract] OR "sub saharan africa"[Title/Abstract] OR "sub saharan africa"[Title/Abstract] OR "Africa"[Title/Abstract] OR "Africa"[MeSH Terms])) AND ((humans[Filter]) AND (english[Filter])) | Results= 4438 |
| **SCOPUS** | anxiety OR "Anxiety Disorders" OR anxi* OR "Anxiety symptoms" OR "Symptoms of anxiety" OR "Generalized anxiety disorder" OR gad OR "posttraumatic stress disorder" OR ptsd OR "Traumatic stress" OR "Post-traumatic stress" OR trauma OR "psychological trauma" OR "Stress disorders"  AND  adult* OR "old age" OR elder*  AND  "Africa South of the Sahara" OR "Sub Saharan Africa" OR "sub-Saharan Africa" OR "Sub-Saharan Africa" OR africa OR angola OR benin OR botswana OR "Burkina Faso" OR "Upper Volta" OR burundi OR cameroon OR "Cape Verde" OR "Central African Republic" OR chad OR comoros OR congo OR "Cote D'Ivoire" OR "Ivory Coast" OR zaire OR "Democratic Republic Of The Congo" OR "French Somaliland" OR djibouti OR "Equatorial Guinea" OR eritrea OR ethiopia OR "Gabonese Republic" OR gabon OR gambia OR "Gold Coast" OR ghana OR guinea OR "Guinea-Bissau" OR kenya OR basutoland OR lesotho OR liberia OR "Malagasy Republic" OR madagascar OR nyasaland OR malawi OR mali OR mauritania OR mauritius OR mayotte OR mozambique OR namibia OR niger OR nigeria OR reunion OR rwanda OR ruanda-urundi OR "Sao Tome & Principe" OR "Sao Tome" OR senegal OR seychelles OR "Sierra Leone" OR somalia OR "South Africa" OR "South Sudan" OR sudan OR swaziland OR eswatini OR "Togolese Republic" OR togo OR uganda OR "United Republic Of Tanzania" OR tanzania OR zambia OR zimbabwe OR rhodesia OR "Africa Eastern" OR "Africa Southern" OR "Africa Western" OR "Africa Central"  *Filters*  -Limit to English  -Limit keywords- Human , Humans  -Excluded *conference papers, letter, book chapter, note, editorial, book, retracted, conference review*  -Excluded countries/territory; *United States, United Kingdom, Australia, Canada, France, Germany, Netherlands, Switzerland, Sweden, Belgium, Norway, China, Spain, India, Israel, New Zealand, Saudi Arabia, Denmark, Brazil, Japan, Thailand, Turkey, Mexico, Finland, Portugal, Hongkong, United Arab Emirates, Pakistan, Lebanon, Austria, Iran, Qatar, South Korea, Peru, Bangladesh, Argentina, Russian Federation, Greece, Romania, Jordan, Hungary, Oman, Philippines, Bulgaria, Vietnam, Czech Republic, Ukraine, Syrian, Arab Republic, Luxemberg, Yemen, Fiji, Haiti, Iceland, Slovakia, Panama, Palestine, Bolivia, Uruguay, Turkiye, Venezuela, Macao, Cuba, North Macadamia, Trinidad and Tobago, Perto Rico, Bahrain, Cambodia, Estonia, Gyana, Kazakhastan, Kiribati, Kuwait, Martinique, Nicaragua, Singapore, Croatia, Poland, Taiwan, Iraq, Malaysia* | Results= 3414 |
| **PSYCINFO** | XB anxiety OR MA anxiety disorders OR XB anxiety disorders OR MA generalized anxiety disorder OR XB anxi* OR XB anxiety symptoms OR XB symptoms of anxiety OR XB GAD OR MA stress disorders, post traumatic OR XB ( (posttraumatic stress disorder) OR (PTSD) OR (traumatic stress) OR (post traumatic stress) OR (trauma) OR (psychological trauma) ) OR MA psychological trauma OR XB stress disorder  AND  MA adult OR XB old age OR XB elder*  AND  MA africa south of the sahara OR XB africa south of the sahara OR XB Sub Saharan Africa OR XB sub-Saharan Africa OR XB Sub-Saharan Africa OR XB Africa OR MA Africa  ***Filters***  -Narrow by language-*English*  -Narrow by methodology- *empirical study, quantitative study, longitudinal study, retrospective study, prospective study, follow up study, literature review, field study* | Results=  341 |
| **CINHAL** | XB anxiety OR MW anxiety disorders OR MW generalized anxiety disorder OR XB ( (anxi*) OR (anxiety symptoms) OR (symptoms of anxiety) OR (GAD) ) OR XB posttraumatic stress disorder OR MW stress disorders, post traumatic OR XB PTSD OR XB traumatic stress OR XB post traumatic stress OR XB ( (trauma) OR (psychological trauma) ) OR MW psychological trauma OR XB stress disorders  AND  MW adult OR XB old age OR XB elder*  AND  MW africa south of the sahara OR XB africa south of the sahara OR XB Sub Saharan Africa OR XB sub-Saharan Africa OR XB Sub-Saharan Africa OR XB Africa OR MW Africa  *Filters*  Filter by language -*English* | Results= 642 |
| **WEB OF SCIENCE** | TS=(("Anxiety" OR "Anxiety Disorders" OR Anxi* OR "Anxiety symptoms" OR "Symptoms of anxiety" OR "Generalized anxiety disorder" OR GAD OR "posttraumatic stress disorder" OR PTSD OR "Traumatic stress" OR "Post-traumatic stress" OR trauma OR "psychological trauma" OR "Stress disorders") AND (Adult* OR "old age" OR elder*) AND ("Africa South of the Sahara" OR "Sub Saharan Africa" OR "sub-Saharan Africa" OR "Sub-Saharan Africa" OR Africa OR Angola OR Benin OR Botswana OR "Burkina Faso" OR "Upper Volta" OR Burundi OR Cameroon OR "Cape Verde" OR "Central African Republic" OR Chad OR Comoros OR Congo OR "Cote D'ivoire" OR "Ivory Coast" OR Zaire OR "Democratic Republic Of The Congo" OR "French Somaliland" OR Djibouti OR "Equatorial Guinea" OR Eritrea OR Ethiopia OR "Gabonese Republic" OR Gabon OR Gambia OR "Gold Coast" OR Ghana OR Guinea OR "Guinea-Bissau" OR Kenya OR Basutoland OR Lesotho OR Liberia OR "Malagasy Republic" OR Madagascar OR Nyasaland OR Malawi OR Mali OR Mauritania OR Mauritius OR Mayotte OR Mozambique OR Namibia OR Niger OR Nigeria OR Reunion OR Rwanda OR "Ruanda-Urundi" OR "Sao Tome & Principe" OR "Sao Tome" OR Senegal OR Seychelles OR "Sierra Leone" OR Somalia OR "South Africa" OR "South Sudan" OR Sudan OR Swaziland OR Eswatini OR "Togolese Republic" OR Togo OR Uganda OR "United Republic Of Tanzania" OR Tanzania OR Zambia OR Zimbabwe OR Rhodesia OR "Africa Eastern" OR "Africa Southern" OR "Africa Western" OR "Africa Central"))  *Filters*  -Language; *English*  -Exclude countries ; *USA, England, Canada, Australia, Germany, France, Netherlands, Switzerland, peoples r china, Italy, Sweden, india, Thailand, spain, Belgium, Saudi arabia, Japan, Norway, brazil, new zealand, Scotland, Denmark, finland, Mexico, Egypt, iran, israel, Pakistan, Greece, Argentina, turkiye, Poland, Jordan, Syria, Iraq, Vietnam, Philippines, Colombia, Indonesia, u arab emirates, south korea, Qatar,Malaysia, Bangladesh, Portugal, Lebanon, Austria, turkey, north Ireland, Ireland, hungary, Russia, Singapore, Cambodia, Guatemala, oman, Ukraine, Gibraltar, Haiti, Luxembourg, Palestine, Romania, yemen, Afghanistan, Barbados, Bulgaria, Croatia, cyprus, Georgia, Iceland, Jamaica, Kuwait, laos, sri lanka, Swaziland, Uruguay, Czech republic*  <https://www.webofscience.com/wos/woscc/summary/3dbfaaf7-0aaa-4e5c-a897-7ee973c7b5c8-016295da29/relevance/1> | Results= 642 |
